# Increased Risk of Central Mesocolic Lymph Node Metastases in BRAF-Mutated Stage I-III Colon Cancer

**DOI:** 10.3390/jcm15072766

**Published:** 2026-04-06

**Authors:** Justas Kuliavas, Kristina Marcinkeviciute, Ieva Vaicekauskaite, Rasa Sabaliauskaite, Augustinas Bausys, Audrius Dulskas, Ugnius Mickys, Rokas Stulpinas, Kestutis Strupas

**Affiliations:** 1 Department of Abdominal and General Surgery and Oncology, National Cancer Centre Branch of Vilnius University Hospital Santaros Klinikos, Santariskiu Str. 1, LT-08406 Vilnius, Lithuania; justas.kuliavas@gmail.com; 2 Faculty of Medicine, Vilnius University, M. K. Ciurlionio Str. 21/27, LT-03101 Vilnius, Lithuania; audrius.dulskas@gmail.com (A.D.); rokas.stulpinas@santa.lt (R.S.); 3 Laboratory of Genetic Diagnostic, National Cancer Institute, Santariskiu Str. 1, LT-08406 Vilnius, Lithuania; ieva.vaicekauskaite@nvi.lt (I.V.); rasa.sabaliauskaite@nvi.lt (R.S.); 4 Laboratory of Experimental Surgery and Oncology, Translational Health Research Institute, Faculty of Medicine, Vilnius University, LT-03101 Vilnius, Lithuania; abpelikanas@gmail.com (A.B.); kestutis.strupas@santa.lt (K.S.); 5 National Center of Pathology, Affiliate of Vilnius University Hospital Santaros Klinikos, P. Baublio Str. 5, LT-08406 Vilnius, Lithuania; ugnius.mickys@santa.lt

**Keywords:** colorectal cancer, lymphadenectomy, BRAF mutation, KRAS mutation

## Abstract

**Background**: The optimal extent of lymphadenectomy in colon cancer (CC) remains controversial. While Complete Mesocolic Excision (CME)/D3 dissection may improve oncological outcomes, the survival benefit appears limited to patients with central lymph node metastases (LNMs). Molecular profiling could help identify patients who may benefit from extended lymphadenectomy. **Methods**: This prospective cohort sub-study of the international T-REX trial included 97 patients with stage I–III CC who underwent curative resection at the National Cancer Centre, Vilnius, Lithuania (2015–2018). Lymph node mapping was performed by anatomical zones, and BRAF and KRAS mutation status in primary tumors was determined by quantitative PCR. Associations between genetic mutations, LNM distribution, and survival outcomes were analyzed. **Results**: A total of 2710 lymph nodes were retrieved from 97 patients, of which 100 (3.7%) were metastatic, and identified in 33 (34.0%) patients. Central LNMs were observed in 5 (5.2%) patients overall but were significantly more frequent among those with BRAF-mutated tumors (30.8%) compared to KRAS-mutated (2.4%) or wild-type (0%) cases (*p* < 0.001). BRAF mutations were associated with increased odds of intermediate (OR 8.1, 95% CI 1.4–45.6) and central (OR 36.8, 95% CI 3.7–366.7) LNMs. Mutation status did not impact overall or disease-free survival. **Conclusions**: BRAF mutations in primary CC are linked to higher rates of intermediate and central LNMs. Patients with BRAF-mutated tumors may benefit from extended lymphadenectomy. Future randomized trials should evaluate biomarker-driven surgical strategies in CC.

## 1. Introduction

Colorectal cancer (CC) is the third most common malignancy worldwide, with incidence steadily increasing over recent decades [1]. Surgery remains the cornerstone of curative treatment, but the optimal extent of lymphadenectomy continues to be debated. The Japanese Society for Cancer of the Colon and Rectum recommends D3 dissection for T3–T4 tumors or node-positive disease to ensure accurate staging and potentially improve oncological outcomes [2]. In Western practice, Complete Mesocolic Excision with Central Vascular Ligation (CME with CVL) has been proposed as an analogous approach [3,4], with some studies suggesting benefits in survival and staging accuracy [3,5]. However, high-level evidence remains inconclusive: the phase III RELARC trial did not demonstrate disease-free survival superiority of CME compared to D2 dissection [6], and only a small proportion of patients (6.7%) harbor metastases in D3 lymph nodes [7]. Moreover, extensive dissections such as CME are technically demanding, may increase perioperative risks, prolong hospitalization, and impact quality of life [8]. Thus, the balance between oncological radicality and surgical safety remains unresolved [5,9,10].

Currently, preoperative imaging modalities such as CT and PET scans can predict lymph node involvement with only moderate accuracy (sensitivity and specificity up to 67%) [11]. A more precise method to identify patients at higher risk of lymph node metastasis could help tailor surgical strategies—reserving extended lymphadenectomy for those most likely to benefit while avoiding unnecessary morbidity in others. Genetic profiling offers a promising avenue for such risk stratification. In papillary thyroid cancer, the presence of a BRAF mutation has been linked to a higher risk of lymph node metastasis, directly influencing surgical decision-making [12]. Similar approaches may be valuable in CC, where mutations in KRAS, NRAS, BRAF, and PIK3CA have been associated with lymph node involvement in preliminary studies [13]. In clinical practice, KRAS and BRAF mutations are among the most frequently observed genetic alterations in CC [14]. Identifying whether these mutations correlate with distinct patterns of nodal spread could enable a more personalized surgical approach—directing extensive lymphadenectomy to genetically high-risk patients while minimizing overtreatment in others. Therefore, the present study aimed to assess the impact of KRAS and BRAF mutations on CC lymph node metastasis, with a particular focus on distribution across different nodal zones.

## 2. Materials and Methods

### 2.1. Study Design and Participants

This is a retrospective sub-study of a previous prospective observational cohort study of the International Cohort Study for Optimal Bowel Resection Extent and Central Radicality for Colon Cancer (T-REX study) [15]. This study included all colon cancer patients enrolled in the T-REX study at the National Cancer Centre (a branch of Vilnius University hospital Santaros Klinikos), Vilnius, Lithuania, who underwent surgery between 2 June 2016 and 31 December 2018. The original inclusion criteria of the T-REX study were applied to this sub-study and included: (1) histologically confirmed colon cancer; (2) tumor stages I–III; (3) potentially curative surgery; and (4) informed consent for data collection. Exclusion criteria were: (1) Tis (mucosal carcinoma), (2) multiple colon cancers, and (3) receipt of preoperative adjuvant therapy. The original T-REX study was approved by the Vilnius Regional Bioethics Committee prior to the enrollment of the first patients. This retrospective sub-study was subsequently approved by the same ethics committee for the retrospective analysis of the previously prospectively collected cohort (approval No. 2019/3-1116-608, including subsequent amendments). It was conducted in accordance with the ethical standards of the Declaration of Helsinki (2013 revision).

### 2.2. Surgery and Lymph Node Mapping

All patients underwent radical colon cancer resection, with the choice of an open or laparoscopic approach left to the operating surgeon’s discretion. The procedure for lymph node mapping has been described previously [16]. Briefly, retrieved LNs were grouped as pericolic, intermediate, or central. Central LNs were defined as those along the superior mesenteric artery (SMA) or superior mesenteric vein (SMV) at the root of the colic artery for right-sided colon cancers and those along the IMA proximal to the origin of the left colic artery (LCA) for left-sided colon cancers. Intermediate lymph nodes were defined as nodes located around the feeding artery or arteries. Pericolic lymph nodes were classified as those located within the following: the primary tumor area, 0 < distance from the tumor (D) ≤ 5 cm, 5 < D ≤ 10 cm, 10 < D ≤ 15 cm, 15 < D ≤ 20 cm, and D > 20 cm. The T-REX study does not regulate the level of lymph node dissection or the bowel resection length [15,16]. Two pathologists performed the lymph node mapping.

### 2.3. Data Collection

Data collection followed the methodology of the original T-REX study [15]. The variables included: patient characteristics (age, sex, height, body weight, and tumor location), treatment characteristics (date of surgery, type of operation, intraoperative marking method, bowel length resected, level of central radicality, operation time, estimated blood loss, postoperative morbidity and mortality, data on adjuvant chemotherapy, and data on disease progression) and pathological examination results (depth of tumor invasion (pT), tumor grade, residual tumor status, circumferential margin involvement, and lymph node metastases distribution) [15]. The long-term follow-up protocol consisted of periodic assessments of CEA levels and imaging studies (computed tomography, ultrasound, and X-ray) performed four times per year initially for 2 years, then biannually and subsequently annually, along with an endoscopic evaluation one year after surgery. For the purpose of this sub-study, the mutation statuses of BRAF and KRAS in the primary tumor were also evaluated, as described below.

### 2.4. KRAS and BRAF Mutation Analysis: DNA Extraction and Quantitative PCR

To assess the impact of KRAS and BRAF mutations on CC lymph node metastasis patterns, DNA was extracted from 97 primary colorectal cancer FFPE tumor samples (5 µm thick) using the GeneJET FFPE DNA Purification Kit (Thermo Scientific, Vilnius, Lithuania), following the manufacturer’s protocol. DNA purity and concentration were measured with a Nanodrop 2000 spectrophotometer (Thermo Scientific, Wilmington, DE, USA), and samples were stored at –80 °C until analysis. Mutation testing was performed by quantitative PCR (qPCR) on an ABI 7500 Real-Time PCR System (Applied Biosystems, Singapore) using the BRAF Codon 600 Mutation Analysis Kit II (V600E/K/D/R/M/G) and the KRAS Mutation Analysis Kit (exons 2–4) (Entrogen, Los Angeles, CA, USA), according to the CE-IVD manufacturer’s instructions. Data were analyzed with 7500 Software v2.3 (Applied Biosystems).

### 2.5. Statistical Analysis

All statistical analyses were conducted using SPSS^®^ version 25.0 (IBM, Armonk, NY, USA). Descriptive statistics were used to describe interval and ratio variables in terms of medians, first quartiles (Q1), and third quartiles (Q3). The normality of data was checked, using the Shapiro–Wilk and Kolmogorov–Smirnov (K–S) tests. Nominal and ordinal variables were described, using frequencies and percentages. The Chi-squared test and t-test were used to evaluate the differences between patient groups. Kaplan–Meier curves and the log-rank test were used to analyze the overall and disease-free survival.

## 3. Results

### 3.1. Baseline Characteristics

In total, 97 CC patients with a median age of 67 (57; 73) years were included in this study. BRAF and KRAS mutations were detected in 13 (13.4%) and 41 (42.3%) patients, respectively. None of the patients had both mutations. The baseline characteristics of these patients are shown in Table 1. BRAF mutations were more common among patients with right-side CC (9 of 39 (23.1%)) vs. left with 4 of 54 (6.9%), *p* = 0.022).

### 3.2. Lymph Node Metastases and BRAF/KRAS Mutations Impact on It

**Figure 1:**
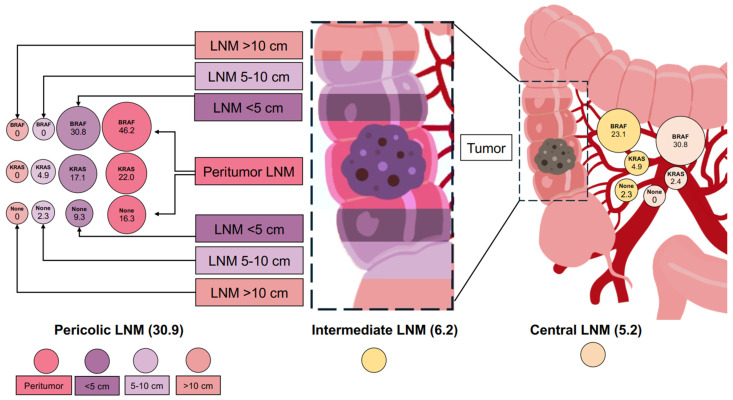
Detailed lymph node metastases map in general cohort and according to detected mutations (created by Canva).

In total, 2710 lymph nodes were retrieved during this study; a total of 100 (3.69%) of them were metastatic, and these lymph node metastases (LNMs) were detected in 33 (34.0%) patients. Detailed mapping of the LNMs revealed that pericolic metastases were the most common, occurring in 30 (30.9%) patients. Within the pericolic region, peritumorous metastases were present in 22 (22.7%) patients. LNMs within the pericolic area up to 5 cm from the tumor were detected in 15 (15.5%) patients, whereas LNMs located 5–10 cm from the tumor were found in 3 (3.1%) patients. No metastases were detected in the pericolic area beyond 10 cm from the tumor. Intermediate level LNMs were found in six (6.2%) patients and central LNMs were present in five (5.2%) patients. Figure 1 shows a detailed mapping of LNMs in the general cohort and according to the detected mutations. Despite the overall low rate of central LNMs in the general cohort, the rate of LNMs in this zone was significantly higher among patients with BRAF-mutated tumors (30.8%) compared to those with KRAS mutations (2.4%) or no mutations (0%) (*p* < 0.001). CCs with BRAF mutations were associated with a higher rate of LNMs (8 (61.5%) vs. 25 (29.8%), *p* = 0.024) compared to CCs without BRAF mutations. Moreover, tumors with BRAF mutations had high odds of LNMs in intermediate (OR:8.1; 95% CI: 1.4–45.6) and central (OR:36.8; 95% CI: 3.7–366.7) LN stations.

### 3.3. Long-Term Outcomes

**Figure 2:**
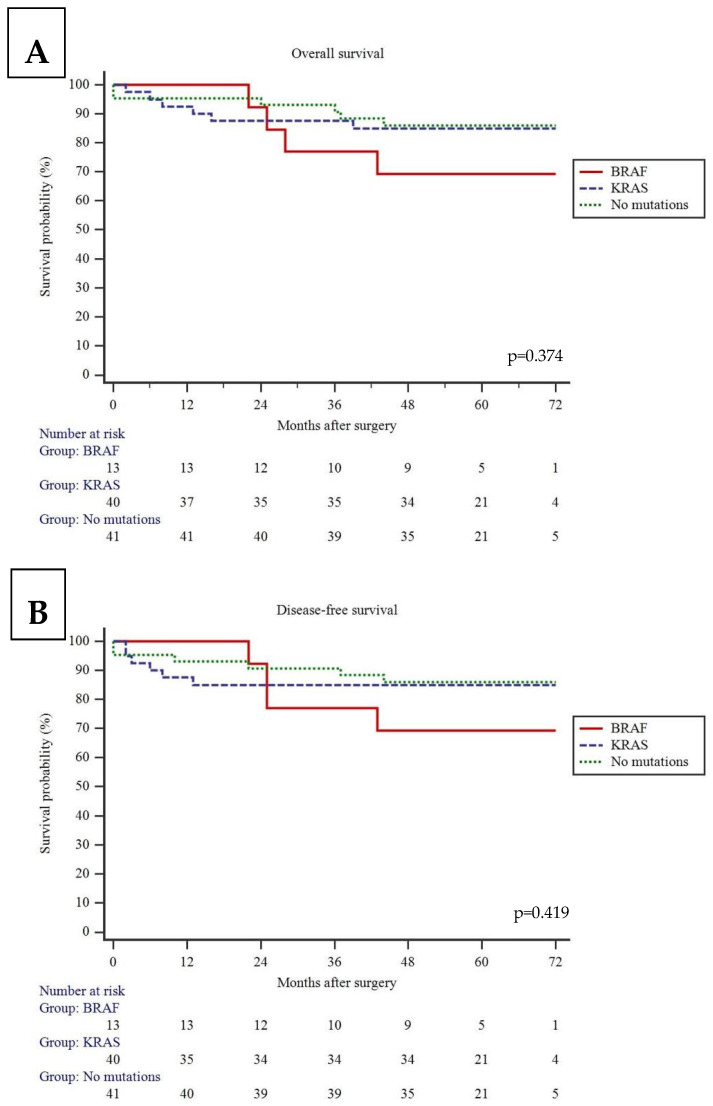
Kaplan–Meier curves. (**A**)—Overall survival (OS). (**B**)—Disease-free survival (DFS).

The median time to follow up was 60 (54; 68) months. A univariate Kaplan–Meier analysis showed similar 5-year overall survival (OS) (85.0% vs. 69.2% vs. 86%, *p* = 0.374) and disease-free survival (DFS) (85.0% vs. 69.2% vs. 86%, *p* = 0.419) among patients with KRAS, BRAF, and no mutation tumors (Figure 2). Additional multivariable Cox regression analysis demonstrated that age and right-sided cancer increased the risk for death and cancer recurrence, while BRAF and KRAS mutations did not (Table 2).

## 4. Discussion

This study demonstrated that BRAF mutations in primary colon cancer tumors are associated with higher rates of lymph node metastasis, including significantly increased odds of metastases at intermediate and central mesocolon nodal levels. However, neither BRAF nor KRAS mutations were confirmed to have a negative impact on the long-term outcomes of patients with CC. The overall incidence of lymph node metastasis was highest in the pericolic region, particularly in the peritumoral area, whereas involvement in intermediate and central stations was only 5–6%. Importantly, no metastases were observed in pericolic nodes located more than 10 cm from the primary tumor.

Specific gene mutations may alter tumor behavior and be associated with lymph node metastasis [17]. BRAF and KRAS are among the most prevalent oncogenes in colon cancer, with KRAS mutations reported in up to 50% of cases [18] and BRAF mutations in up to 15% [19]. In the present study, we observed similar rates, with 42% of patients harboring KRAS mutations and 13% with BRAF mutations. BRAF-positive tumors are known to exhibit more aggressive behavior [20,21,22,23]. In this study, we showed that among patients with non-metastatic CC, BRAF mutations are associated with an increased likelihood of lymph node metastases in intermediate and central mesocolon zones. This finding may have important clinical implications for risk stratification and surgical planning, as patients with BRAF mutations could potentially benefit from D3 lymphadenectomy. Extended lymphadenectomy in CC surgery remains a topic for ongoing debates. On the one hand, the recent results from the RELARC study were negative and failed to show improved long-term outcomes with CME dissection [6]. On the other hand, numerous non-randomized studies showed D3 or CME advantage for survival in patients with T3/T4 tumors [24]; elderly patients with stage II-III cancer [25]; patients with right-sided tumors [26]; or patients with only a stage III tumor [10]. Such notable differences between the findings of non-randomized studies and RCT results are hard to explain, but several factors may be considered. First, there is variation in terminology in the literature. D3 dissection and CME have often been used interchangeably, although they differ in certain aspects. CME may involve resection of some extra-mesenteric lymph nodes [27], whereas D3 dissection specifically focuses on the removal of central nodes [28]. More recently, the removal of central lymph nodes has been regarded as the principal requirement for CME in terms of central radicality [10], making D3 dissection and CME largely comparable. Second, there is no widely accepted standard for what constitutes a conventional colon resection or D2 dissection, as the medial border of D2 lymph node dissection is not precisely defined in the literature. Thus, the lack of a unified definition for conventional/D2 surgery can make comparisons between studies difficult and may lead to misleading interpretations of treatment outcomes. Third, and perhaps the most important concern regarding the routine implementation of D3 dissection, is that it primarily benefits only patients with metastases in central lymph nodes. The largest dataset to date, from the T-REX study, showed that only about 3% of patients with non-metastatic colon cancer had metastases in these stations [16]. In the present study, conducted as part of the T-REX study at a Western site, the rate of central lymph node metastases was slightly higher, exceeding 5%. Nevertheless, this proportion remains low. When considered alongside evidence that extended lymphadenectomy may increase the risk of severe intraoperative complications, such as vascular injury [29], as well as higher postoperative morbidity [8,30], routine implementation of D3 dissection appears difficult to justify. However, the present study showed 30.8% LNM rate in central nodes among patients with BRAF mutations and markedly increased odds of central lymph node metastases in patients with BRAF-mutated tumors. This suggests that not only is it likely that patients with BRAF-mutated tumors may benefit from D3/CME colon surgery, but also that future randomized trials comparing conventional and extended lymphadenectomy could yield positive results if designed to be personalized and biomarker-driven, specifically targeting patients with BRAF-mutated tumors.

In this study, we also found an increased likelihood of BRAF mutations in right-sided tumors. This aligns with the serrated neoplasia pathway, the typical route for right-sided CC, in which a BRAF mutation is a hallmark event, driving progression from sessile serrated polyps to carcinoma [31]. Anatomically, the right colon develops from the midgut, while the left colon originates from the hindgut [32]. As a result, the right- and left-sided colon tissues are molecularly distinct, with right-sided genes more prone to age-related methylation and functional impairment [33]. Based on our data, BRAF and KRAS mutations did not have a direct impact on overall mortality. While some studies have reported a significant association between these mutations and all-cause mortality [34], others indicate that the prognostic impact depends on microsatellite status, which is consistent with our findings. Specifically, microsatellite-unstable (MSI) CC shows no increase in mortality despite BRAF or KRAS mutations, whereas microsatellite-stable (MSS) CC with these mutations is associated with a worse prognosis [35,36].

The present findings should be interpreted with some caution. A major limitation of this study is a lack of formal surgical quality control, which makes it uncertain whether all relevant lymph nodes were consistently resected and analyzed. In addition, the T-REX study did not require extended lymphadenectomy, potentially influencing the observed rates of central lymph node metastasis. The single-center design and relatively small sample size may limit generalizability, while the absence of data on coexisting mutations (ex.: no MSI status, NRAS/PIK3CA, etc.) could affect the interpretation of the associations with BRAF and KRAS. Despite these limitations, our findings provide valuable insight into the relationships between tumor location, molecular markers, and lymph node metastasis in colorectal cancer.

## 5. Conclusions

This study demonstrated that BRAF mutations in primary colon cancer tumors are associated with higher rates of lymph node metastasis, including increased odds of metastases at intermediate and central nodal levels. Patients with BRAF-mutated tumors may be candidates for extended lymohanodectomy.

## Figures and Tables

**Table 1:** Baseline characteristics of study cohort and patients with *BRAF* and *KRAS* mutations.

		Cohort (*n* = 97)	*BRAF*			*KRAS*		
			*BRAF*+ (*n* = 13)	*BRAF*− (*n* = 84)	*p* Value	*KRAS*+ (*n* = 41)	*KRAS*− (*n* = 56)	*p* Value
Age (median; Q1, Q3)		67 (57; 73)	69 (62; 74)	67 (56; 72)	0.225	67 (56; 72)	67 (57; 73)	0.591
Gender (*n*; %)	Male	45 (46.4%)	3 (23.1%)	42 (50.0%)	0.081	18 (43.9%)	27 (48.2%)	0.674
	Female	52 (53.3%)	10 (76.9%)	42 (50.0%)		23 (56.1%)	29 (51.8%)	
CCI (median; Q1, Q3)		4 (3; 5)	3 (3; 3)	4 (3; 5)	0.522	4 (3; 5)	4 (3; 5)	0.594
pT	T1–2	21 (21.6%)	0 (0%)	21 (25.0%)	0.065	9 (22.0%)	12 (21.4%)	0.951
	T3–4	76 (78.4%)	13 (100%)	63 (75.0%)		32 (78.0%)	44 (78.6%)	
pN	N0	64 (66.0%)	5 (38.5%)	59 (70.2%)	0.024	27 (65.9%)	37 (66.1%)	0.982
	N+	33 (34.0%)	8 (61.5%)	25 (29.8%)		14 (34.1%)	19 (33.9%)	
Pathological stage	I	19 (19.0%)	0 (0%)	19 (22.6%)		8 (19.5%)	11 (19.6%)	0.984
	II	44 (45.4%)	5 (38.5%(	39 (46.4%)		19 (46.3%)	25 (44.6%)	
	III	34 (35.1%)	8 (61.5%)	26 (31.0%)		14 (34.1%)	20 (35.7%)	
Tumor localization	Right-side tumors	39 (59.8%)	9 (69.2%)	30 (35.7%)	0.022	15 (36.6%)	24 (42.9%)	0.534
	Caecum	7 (7.2%)	2 (15.4%)	5 (6.0%)		3 (7.3%)	4 (7.1%)	
	Ascending colon	23 (23.7%)	7 (53.8%)	16 (19.0%)		7 (17.1%)	16 (28.6%)	
	Hepatic flexure	5 (5.2%)	0 (0%)	5 (6.0%)		2 (4.9%)	3 (5.4%)	
	Proximal transverse colon	4 (4.1%)	0 (0%)	4 (4.8%)		3 (7.3%)	1 (1.8%)	
	Left-side tumors	58 (40.2%)	4 (30.8%)	54 (54.3%)		26 (63.4%)	32 (57.1%)	
	Distal transverse colon and splenic flexure	12 (12.4%)	2 (15.4%)	10 (11.9%)		4 (9.8%)	8 (14.3%)	
	Descending colon	7 (7.2%)	1 (7.7%)	6 (7.1%)		5 (12.2%)	2 (3.6%)	
	Sigmoid colon	24 (24.7%)	1 (7.7%)	23 (27.4%)		10 (24.4%)	14 (25.0%)	
	Rectosigmoid junction	15 (15.5%)	0 (0%)	15 (17.9%)		7 (17.1%)	8 (14.3%)	
Median number of retrieved LN		26 (20; 34)	31 (26; 39)	25 (19; 34)	0.056	25 (19; 34)	27 (20; 36)	0.879
Surgical approach	Open	75 (77.3%)	11 (84.6%)	64 (76.2%)	0.500	32 (78.0%)	43 (76.8%)	0.883
	Minimally invasive	22 (22.7%)	2 (15.4%)	20 (23.8%)		9 (22.0%)	13 (23.2%)	
Type of surgery	Right colectomy	37 (38.1%)	9 (69.2%)	28 (33.3%)	0.063	14 (34.1%)	23 (41.1%)	0.775
	Transversal colectomy	3 (3.1%)	0 (0%)	3 (3.6%)		2 (4.9%)	1 (1.8%)	
	Left colectomy/sigmoidectomy	40 (41.2%)	4 (30.8%)	36 (49.2%)		17 (41.5%)	23 (41.1%)	
	Rectal resection	17 (17.5%)	0 (0%)	17 (20.2%)		8 (19.5%)	9 (16.1%)	
Postoperative complications	Yes	18 (18.6%)	1 (7.7%)	17 (20.2%)	0.451	8 (19.5%)	10 (17.9%)	0.836
	No	79 (81.4%)	12 (92.3%)	67 (79.8%)		33 (80.5%)	46 (82.1%)	

**Table 2:** Multivariable Cox regression analysis for overall and disease-free survival.

Variable	Category	Overall Survival		Disease-Free Survival	
		HR (95% CI)	*p*-Value	HR (95% CI)	*p*-Value
Genetic mutations in tumor	No mutations	1 (Reference)		1 (Reference)	
	*BRAF* mutations	1.20 (0.30–4.81)	*0.789*	1.14 (0.40–4.96)	*0.847*
	*KRAS* mutations	1.54 (0.43–5.47)	*0.497*	1.41 (0.40–4.96)	*0.590*
Age		1.13 (1.05; 1.21)	0.001	1.12 (1.05–1.20)	0.001
Stage of the disease	I	1 (Reference)		1 (Reference)	
	II	0.71 (0.12–4.11)	*0.705*	0.74 (0.12–4.29)	*0.743*
	III	3.05 (0.60–15.49)	*0.178*	3.05 (0.60–15.45)	*0.178*
Tumor location	Left-side tumor	1 (Reference)		1 (Reference)	
	Right-side tumor	6.58 (1.84–23.49)	*0.004*	5.72 (1.67–19.54)	*0.005*
Gender	Female	1 (Reference)		1 (Reference)	
	Male	3.09 (1.02–9.39)	*0.046*	2.85 (0.95–8.58)	*0.061*

## Data Availability

Data are available on request due to restrictions.

## Abbreviations

The following abbreviations are used in this manuscript:

CC	Colon cancer
CME	Complete Mesocolic Excision
LNM	Lymph node metastases
CVL	Central Vascular Ligation
